# Managing cow's milk protein allergy during the 2022 formula shortage: decision-making among pediatric healthcare providers

**DOI:** 10.3389/falgy.2024.1359103

**Published:** 2024-05-22

**Authors:** Lea V. Oliveros, Jerry M. Brown, Abigail L. Fabbrini, Andrew A. Farrar, Luke Lamos, Jared Florio, Jesse Beacker, Jessica V. Baran, Michael J. Wilsey

**Affiliations:** ^1^ Office of Medical Education, Alabama College of Osteopathic Medicine, Dothan, AL, United States; ^2^ Office of Medical Education, Florida Atlantic University Charles E. Schmidt College of Medicine, Boca Raton, FL, United States; ^3^ Office of Medical Education, Kansas City University College of Osteopathic Medicine, Kansas City, MO, United States; ^4^ Department of Pediatrics, University of South Florida Morsani College of Medicine, Tampa, FL, United States

**Keywords:** amino acid formula, formula shortage, formula recall, infant nutrition, cow’s milk protein allergy, ZS Moments

## Abstract

**Introduction:**

Cow's milk protein allergy (CMPA) affects 2%-7% of infants and is managed with hypoallergenic formulas. The 2022 recalls of infant formulas due to factors including contamination led to specialty formula shortages, highlighting CMPA management challenges. Understanding healthcare providers' (HCPs) decision-making in transitioning to alternative formulas during shortages is crucial. Limited attention has been given to how pediatric physicians make these choices.

**Methods:**

This study utilized US HCPs' de-identified survey data to assess driving factors when switching extensively hydrolyzed formulas during shortages.

**Results:**

104 eligible HCPs participated, including general pediatrics, pediatric allergy/immunology, and pediatric gastroenterology specialists. Safety, tolerability, and efficacy were identified as top factors for switching formulas. Formula 1 was considered well-tolerated, patient-accepted, and safe by all HCPs. Most expressed strong belief in Formula 1's safety and effectiveness.

**Discussion:**

Findings inform CMPA management during shortages, offering guidance to HCPs for suitable formula selection and enhanced infant care.

## 1 Introduction

The global supply chain disruption resulting from the COVID-19 pandemic led to shortages of many consumer goods, including infant formula, beginning in 2020 (1). The shortage was compounded when, starting in February 2022, recalls of multiple infant formulas and the shutdown of a major formula manufacturing plant led to greatly limited access to specialty formulas. Such formulas, including hypoallergenic formulas, are often necessary for infants inflicted with a variety of conditions, including food allergies, GI issues, premature birth, and infant colic.

A common food allergy in infancy, cow's milk protein allergy (CMPA) is a significant health concern among infants, with a 2%–7% incidence within the first 12 months of life (2, 3). The allergic reaction to cow's milk protein may be IgE- or non-IgE-mediated and can lead to various symptoms and health complications, burdening families with associated healthcare costs, altered quality of life, and future illnesses (4). Treatment for infants with CMPA who are breastfed involves the elimination of cow's milk from the mother's diet for 2–4 weeks, as well as the use of a hypoallergenic formula (1, 5–7). The use of hypoallergenic formulas in the short- and long-term management of CMPA symptoms is well documented (8–11). Hypoallergenic formulas are categorized based on the degree of protein hydrolysis. The extensively hydrolyzed formula contains whey or casein proteins broken down into small peptides, allowing babies intolerant to whole cow's milk proteins to consume the formula without reacting to most cases. The American Academy of Pediatrics recommends extensively hydrolyzed formulas (eHF) as the first-line formula choice. Still, for more severe cases of CMPA where eHF is ineffective, amino acid formulas (AAF) should be used (1, 5).

Infants who are unable to receive hypoallergenic formulas are, therefore, at risk for varying degrees of complications associated with CMPA, including diarrhea from inflammatory disruption of the brush border, failure to thrive due to malabsorption, and allergic reactions due to cross-contamination or accidental consumption (2). The formula shortage introduced unprecedented challenges in managing infants with CMPA, including the need to substitute or recommend different formulas for patients, exacerbating clinicians' already limited access to hypoallergenic formulas while addressing CMPA (1). This scarcity underscored the need for understanding how pediatric HCPs navigate formula selection during such shortages. Addressing the knowledge gap surrounding how pediatric HCPs make decisions on formula selection in CMPA during a formula shortage is essential for improving clinical management and ensuring the provision of appropriate and safe alternatives for infants with CMPA.

This study conducts a cross-sectional analysis of de-identified survey data collected from pediatric HCPs across the United States, focusing on the decision-making factors influencing the selection of extensively hydrolyzed formulas (eHF) during the 2022 national formula shortage. Recognizing the heightened availability of eHF-1 amid this crisis, we hypothesized that HCPs would favor the most accessible eHFs, reflecting a strategic response to the unprecedented challenges posed by the shortage. Our investigation aims to deepen the understanding of the clinical decision-making process guiding pediatric HCPs and to contribute to developing more robust strategies for managing CMPA amidst potential future shortages. Through this analysis, we strive to improve clinical management practices and ensure the provision of safe nutritional solutions for infants with CMPA during challenging formula scarcity.

## 2 Materials and methods

### 2.1 Study objective

We conducted a cross-sectional analysis of de-identified survey data collected from pediatric HCPs in the United States to assess their satisfaction levels when switching to another eHF during the formula shortage and recall crisis that affected patients clinically diagnosed with CMPA.

### 2.2 Study design and participants

Pediatric HCPs included in the study were required to have at least 2 years of experience in a clinic-based practice setting, specialization in general pediatrics, pediatric gastroenterology, or pediatric allergy/immunology, treated at least 7 newly diagnosed CMPA patients between 0 and 24 months old in the last month, and have switched from an initial eHF to an alternative eHF, *Nutramigen,* during the formula shortage period from January 2022 to November 2022. For the purposes of this study, Nutramigen® (*Mead Johnson Nutrition, Evansville, IN, USA),* Alimentum® (*Abbott Nutrition, Abbott Park, IL, USA*), and Extensive HA® (*Gerber Products Co., Arlington, VA, USA*) will be referred to as EHF-1, EHF-2, and EHF-3, respectively. This study received exempt status from the Institutional Review Board (IRB), indicating that it met the criteria for exemption from full IRB review.

### 2.3 Data collection and variables

This study gathered data from pediatric HCPs regarding their gender, specialty, clinical practice setting, years of experience, and the average number of weekly patients, including those with CMPA. Survey questions were developed based on a literature review and discussions with healthcare providers who manage patients with CMPA. The survey was administered online through the ZS Moments platform, by ZS Associates, and comprised 28 questions, which took about 10–15 min to complete. The ZS Moments mobile-based application allows pediatric HCPs to record data securely and accurately in real-time on a user's mobile device. The survey aimed to capture information on pediatric HCPs' demographics and details of the formula switch, as well as pediatric HCPs' perceptions of formula availability, ease of obtaining the new formula, impact of the formula switch on patient health, and the financial implications of the switch. A “high” rating was determined to be a rating of 8–10 on a scale of 1–10. Our study defines “tolerability” as patients' ability to consume eHF without adverse effects, and “safety” as the absence of allergic reactions, ensuring eHF meets hypoallergenic standards. “Efficacy” is the formula's success in meeting nutritional needs and managing CMPA symptoms, while “patient success” tracks observed improvements in infant health and symptom resolution following eHF use. These terms help frame our evaluation of eHF-1 in managing CMPA.

### 2.4 Statistical analysis

The collected data were summarized using descriptive statistics, and chi-square tests were utilized to investigate the association between demographic variables and formula switch details. Logistic regression analysis was performed to identify factors associated with difficulty obtaining the new formula and an increase in the financial burden. Statistical significance was determined at *p* < 0.05. The data analysis was conducted using the SPSS® analytics software (International Business Machines Corporation®, Armonk, NY, USA).

## 3 Results

### 3.1 Study participants

Our data analysis included a total of 104 pediatric HCPs, all of whom were physicians with specialties in general pediatrics (*n* = 86), pediatric allergy/immunology (*n* = 17), and pediatric gastroenterology (*n* = 1).

### 3.2 EHF-1 switching and preferences

**Figure 1:**
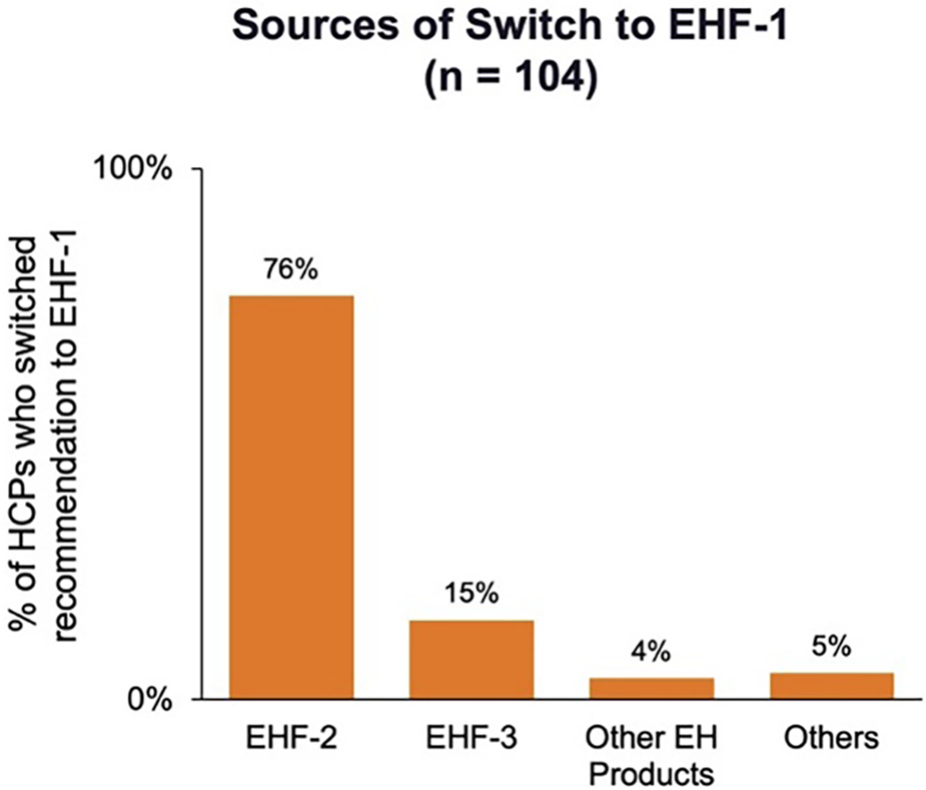
Sources of switch for pediatric HCPs who were using another formula and switched their recommendation to EHF-1.

**Figure 2:**
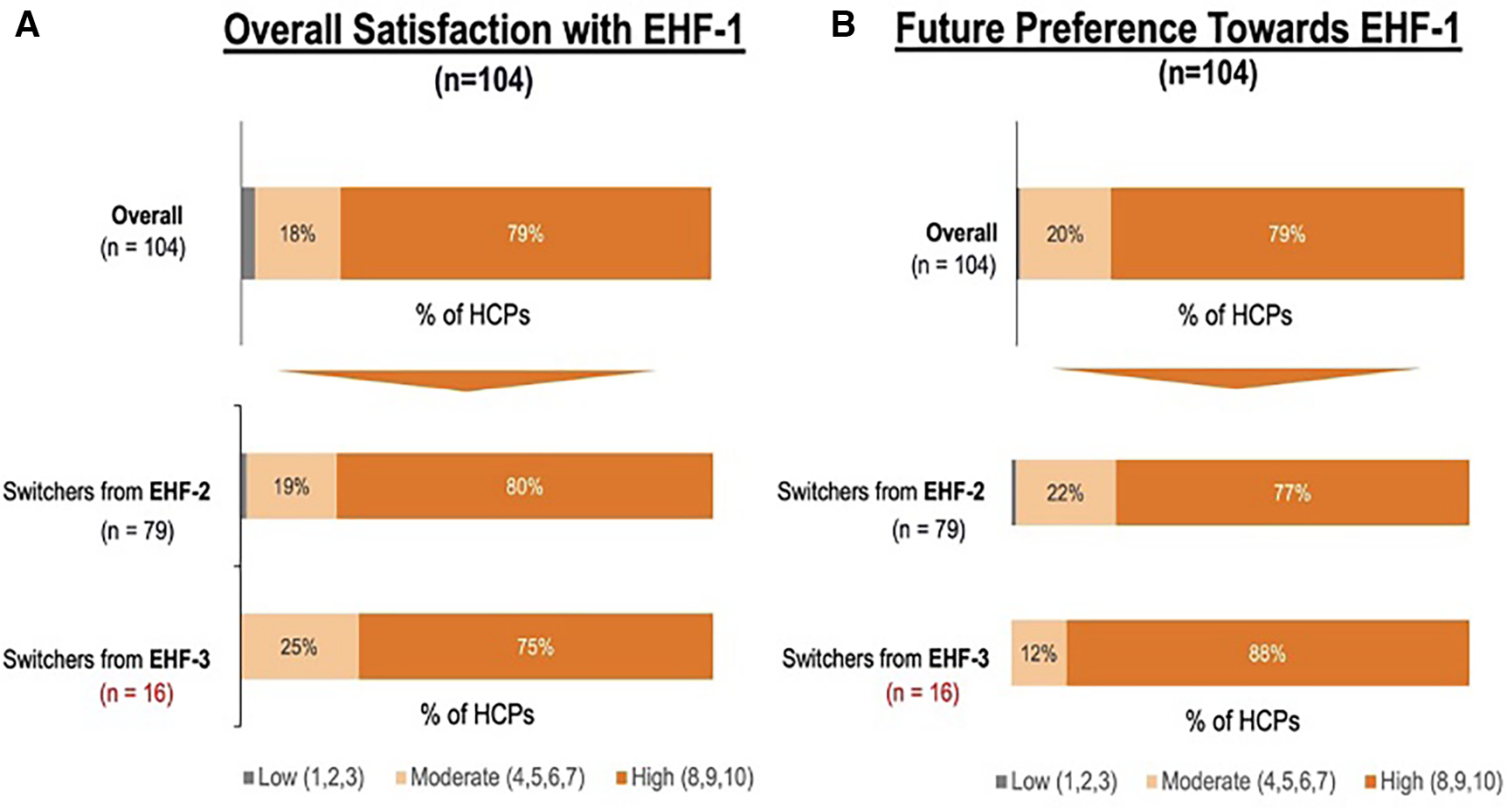
(**A**) percent of pediatric HCPs reporting a high perceived treatment quality of formula 1 after switching from another extensively hydrolyzed formula. (**B**) Percent of pediatric HCPs reporting a continued preference for EHF-1 after the storage overall and broken down by source of switch.

**Figure 3:**
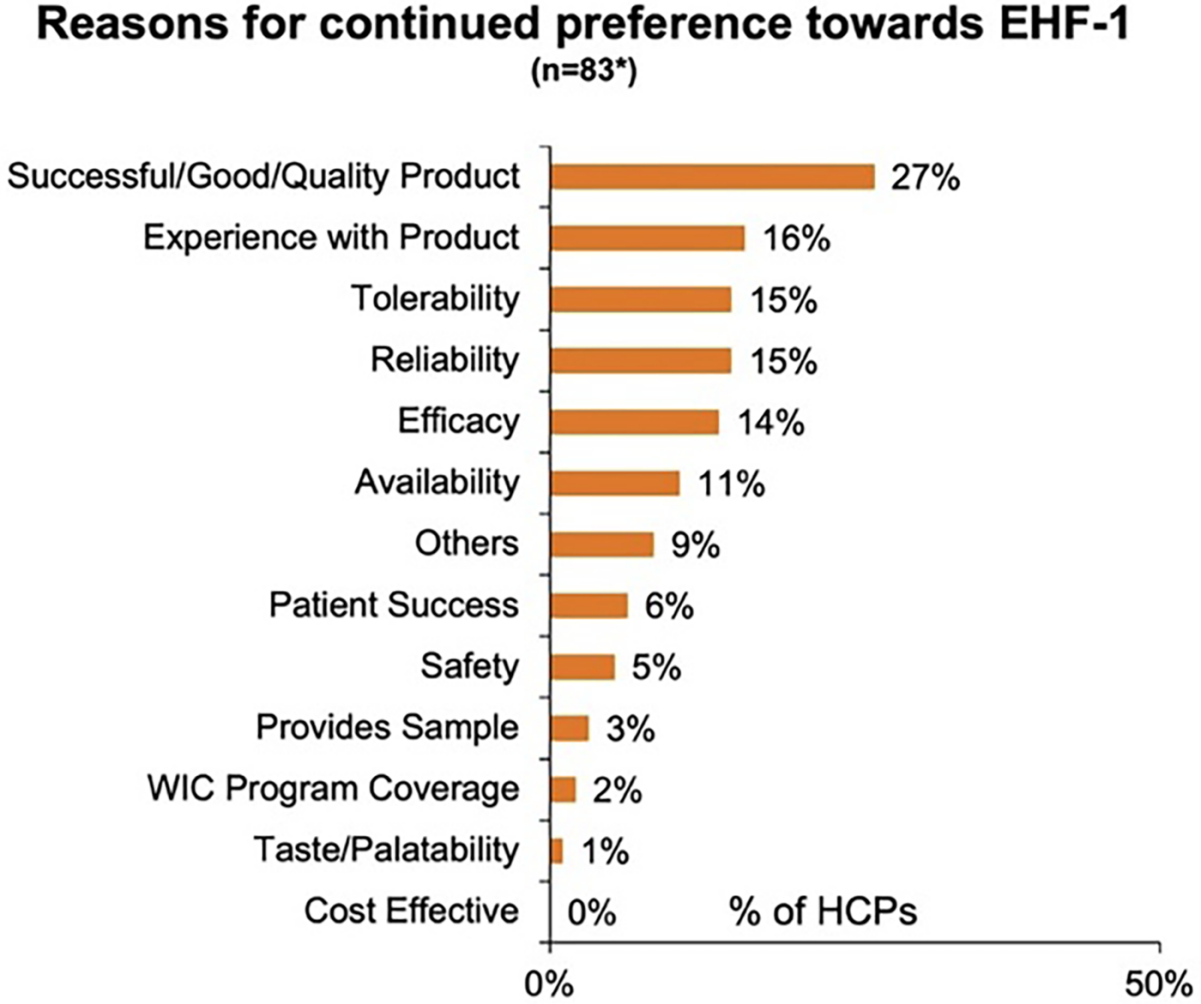
Reasons for continued preference towards EHF-1 as perceived by pediatric HCPs. *Includes responses from all pediatric HCPs rating 8, 9, 10, in future preference.

**Figure 4:**
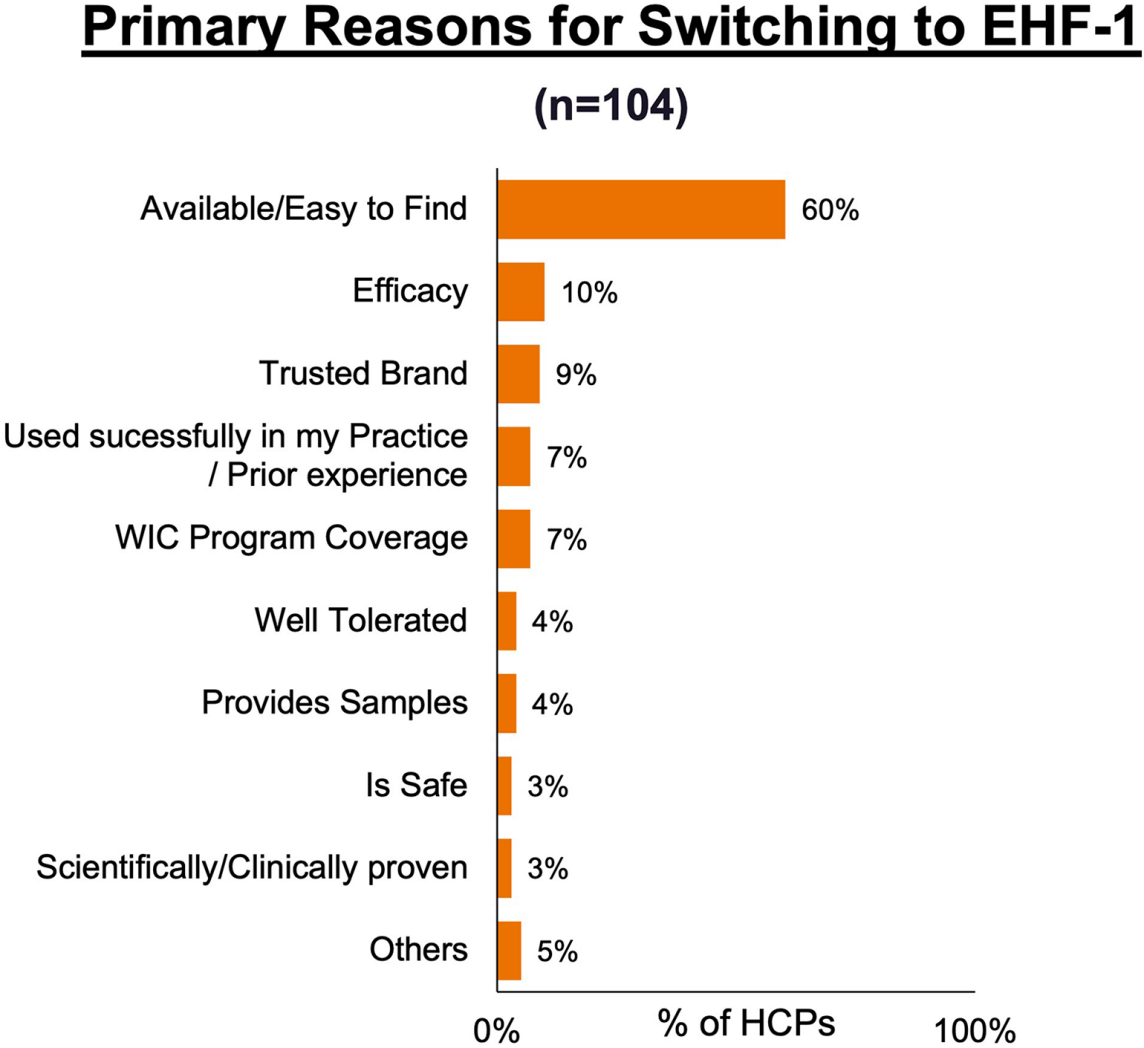
Primary reasons HCPs reported for switching formulas during the formula shortage.

Figure 1 depicts the proportion of patients who switched to EHF-1 initially on EHF-2, EHF-3, or another infant formula. Tolerability (91%), safety (91%), and availability (89%) were the three driving factors most frequently rated as “highly important” to healthcare providers during the formula shortage. When surveyed on perceived performance, pediatric HCPs most frequently rated EHF-1 highly for safety (84%), trust (79%), and efficacy (73%). The most frequent primary reasons reported by pediatric HCPs for switching to EHF-1 were availability (60%), efficacy (10%), and reputation (9%). Tables 1A–1C depicts the percentage of pediatric HCPs that rated the formulas “highly” for each factor within each specialty. Figure 2 illustrates the overall perception of EHF-1 treatment quality after switching and the percentage of pediatric HCPs who reported a continued preference for EHF-1 after switching. Figure 3 depicts reported reasons for pediatric HCP's continued preference for EHF-1 after the shortage. Figure 4 depicts the primary reasons for switching formulas as reported by HCPs.

**Table 1A:** Percentage of HCPs across specialties that rated formulas “highly” for given factors when using EHF-1.

	Cost	Efficacy	Tolerability	Safety	Availability
General pediatrics	35%	80%	74%	86%	59%
Pediatric allergy/immunology	22%	56%	56%	72%	39%
Pediatric gastroenterologist	0%	0%	0%	100%	100%

**Table 1B:** Percentage of HCPs across specialties that rated formulas “highly” for given factors when using EHF-2.

	Cost	Efficacy	Tolerability	Safety	Availability
General pediatrics	31%	70%	68%	74%	23%
Pediatric allergy/immunology	27%	36%	36%	54%	27%
Pediatric gastroenterologist	0%	100%	0%	0%	100%

**Table 1C:** Percentage of HCPs across specialties that rated formulas “highly” for given factors when using EHF-3.

	Cost	Efficacy	Tolerability	Safety	Availability
General pediatrics	66%	89%	78%	89%	56%
Pediatric allergy/immunology	43%	72%	86%	100%	28%
Pediatric gastroenterologist	–	–	–	–	–

## 4 Discussion

**Figure 5:**
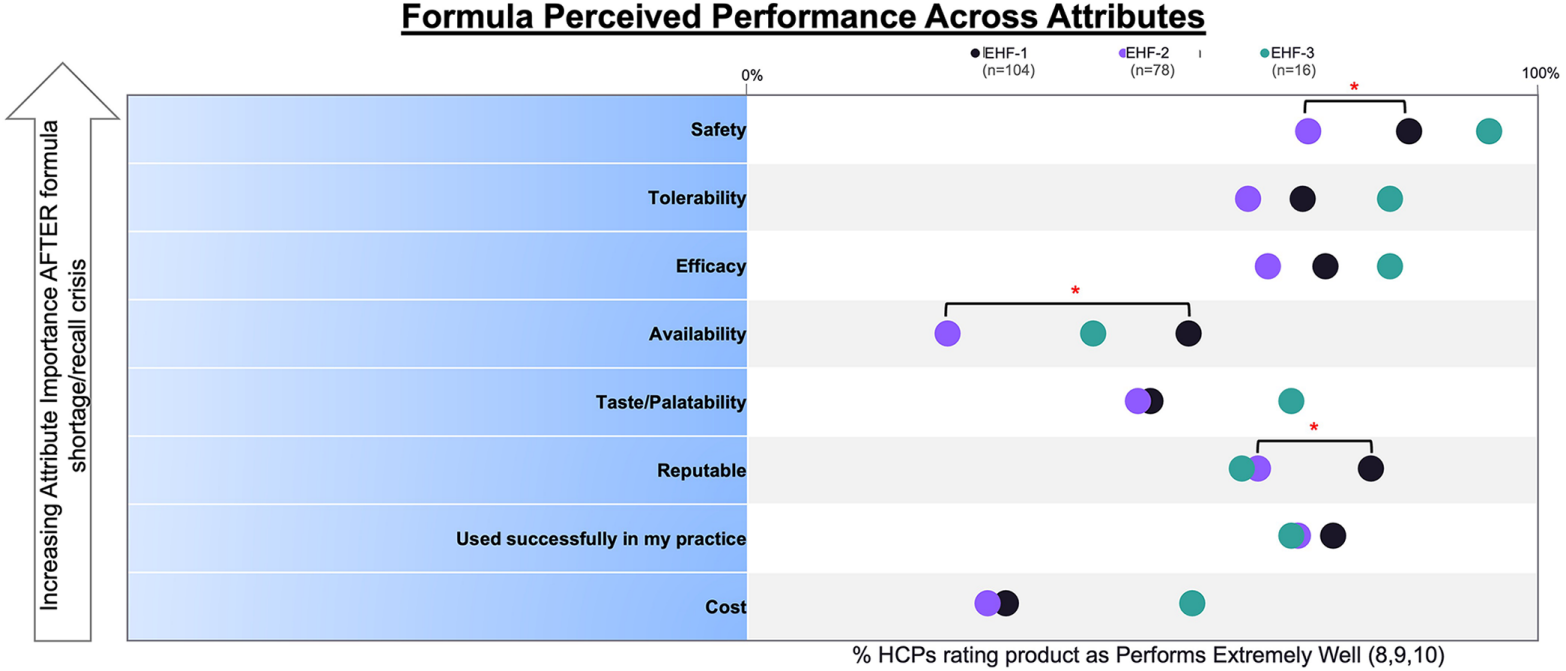
Percent of HCPs who rated each formula “highly” across given attributes.

We conducted a cross-sectional analysis using de-identified survey data from pediatric HCPs in the United States to determine the driving factors in the clinical decision-making process for formula management and when transitioning CMPA patients to alternative eHFs before and during a national shortage. Our results indicate that pediatric HCPs predominantly chose Formula 1 based on its perceived safety, tolerability, and efficacy. Furthermore, there was a significant difference (*p* < 0.05) in perceived safety and availability when comparing EHF-1 and EHF-2, as depicted in Figure 5.

The COVID-19 pandemic has exposed existing inequities in public health infrastructures. In addition, the pandemic sparked worldwide changes in how HCPs diagnose and treat several conditions, including CMPA. All oral food challenges were recommended to be delayed unless a critical acute nutritional need for introducing a key nutrient was indicated. One study found that although more than half the patients with a food allergy were not adherent to their telemedicine appointments, those who were younger or had CMPA were more likely to adhere to those appointments. Formula shortages began during this period and were further exacerbated by the 2022 infant formula shortage.

The unprecedented nature of the 2022 infant formula shortage meant clinicians and parents were met with new challenges when managing CMPA. Some coping strategies used by parents included driving to multiple stores, turning to local milk banks, and receiving donor breast milk (12). Another survey of 863 parents found that 77.4% believed milk banking to be a viable option for addressing the formula shortage (13). While these methods may be acceptable short-term solutions for healthy infants, those with CMPA must rely on breast milk or formula free of cow's milk proteins, requiring additional consideration and limiting the ability to find alternatives (14, 15). Organizations such as the North American Society for Pediatric Gastroenterology, Hepatology, and Nutrition (NASPGHAN) and state Women, Infants, & Children (WIC) offices have come together to form a readily available list of recalled formulas and provide specific alternatives for families and pediatric HCPs (16, 17). NASPGHAN suggested all three formulas surveyed in our study as safe alternative formulas for infants (17), though we found clinicians perceived EHF-1 to be more readily available during the formula shortage.

Although certain studies have examined the reasons behind formula shortages, there remains a paucity in the literature regarding alternative formulas and the factors influencing the decision-making process of pediatric HCPs (18, 19). One article emphasized the need for more robust policies grounded in reproductive justice, aiming to provide greater support to breastfeeding families and establish elevated standards for the safety and production of infant formula (20). The American Academy of Pediatrics reported alternative options during the formula shortage, providing valuable resources for concerned families, such as contacting state WIC and teaching them how to transition to an alternative infant formula (20). However, the article omitted a detailed explanation for choosing these alternatives and overlooked the crucial factors influencing the decision-making process. In a separate study, Abrams and Duggan (2022) observed a prevailing confusion and disorder among families and pediatric HCPs regarding formula marketing, primarily stemming from insufficient substantiation of health benefits claims (18). These implications can render families and clinicians unable to make informed decisions regarding alternative formulas. The World Allergy Organization's Diagnosis and Rationale for Action against Cow's Milk Allergy 2010 guidelines for selecting a replacement formula thoroughly examine and compare different types of hypoallergenic formulas: AAF, eHF, soy formula, rice extensively hydrolyzed formula, soy hydrolyzed formula, and other types of mammal's milk (5). While this study provides valuable information for pediatric HCPs, it is worth emphasizing that the guidelines were developed without considering the possibility of formula shortages. Additionally, the guidelines primarily focus on the specific symptoms elicited when transitioning a patient to an alternative formula rather than why the clinician made the alternative selection.

To our knowledge, this is the first study in the United States examining the clinician perspectives towards selecting infant formula in infants with CMPA during the 2022 infant formula shortage. Understanding the driving factors clinicians use when selecting a hypoallergenic formula during a shortage contributes to the little existing knowledge regarding managing CMPA during formula shortages. The recall of multiple formula brands and subsequent limited access to specialty formulas posed a critical issue in pediatric healthcare.

It is essential to acknowledge the potential limitations of our study. Firstly, our analysis relied on de-identified survey data, possibly introducing response bias. The representativeness of the surveyed pediatric HCPs and generalizability of the findings to the entire population of healthcare professionals may be limited as HCPs included were limited to pediatricians practicing in an office-based clinical setting. Additionally, the study's cross-sectional design restricts our ability to establish causal relationships.

Despite these limitations, this study highlights the impact of limited access to specialty formulas on clinicians' preferences in the care of CMPA patients. It underlines the need for improved strategies to ensure the availability of suitable alternatives. Our study provides insight into the decision-making process and driving factors associated with formula choice and when switching CMPA patients to alternative formulas during shortages. Our study found that formula tolerability, safety, and availability were the most important factors guiding pediatric HCP choice in formula during the shortage. While pediatric HCPs considered EHF-1 as safe and tolerable as alternative formulas, it was also perceived to be significantly easier to find during the shortage, with availability being the main reason for switching for most pediatric HCPs. These findings contribute to the limited body of literature surrounding CMPA management during a severe formula shortage, potentially guiding healthcare providers in making informed decisions and assisting the overall management of patients with CMPA during formula shortages. However, additional studies are needed to better understand the impact of formula shortage on pediatric HCPs.

## Data Availability

The raw data supporting the conclusions of this article will be made available by the authors, without undue reservation.
